# Implications of germination tolerances on invasion potential of *Arthraxon hispidus*

**DOI:** 10.1371/journal.pone.0303638

**Published:** 2024-06-04

**Authors:** Michael C. Beall, Jacob N. Barney, Gregory E. Welbaum, J. Leighton Reid

**Affiliations:** School of Plant and Environmental Sciences, Virginia Tech, Blacksburg, Virginia, United States of America; La Trobe University - Bundoora Campus: La Trobe University, AUSTRALIA

## Abstract

*Arthraxon hispidus* is an introduced, rapidly spreading, and newly invasive grass in the eastern United States, yet little is known about the foundational biology of this aggressive invader. Germination responses to environmental factors including salinity, pH, osmotic potential, temperature, and burial depth were investigated to better understand its germination niche. Seeds from six populations in the Mid-Atlantic US germinated 95% with an average mean time to germination of 3.42 days of imbibition in the dark at 23°C. Germination occurred across a temperature range of 8–37°C and a pH range of 5–10 (≥83%), suggesting that neither pH nor temperature will limit germination in many environments. *Arthraxon hispidus* germination occurred in high salinity (342 mM NaCl) and osmotic potentials as low as -0.83MPa. The NaCl concentration required to reduce germination by 50% exceeded salinity concentrations found in soil and some brackish water saltmarsh systems. While drought adversely affects *A*. *hispidus*, 50% germination occurred at osmotic potentials ranging from -0.25 to -0.67 MPa. Given the climatic conditions of North America, drought stress is unlikely to restrict germination in large regions. Finally, emergence greatly decreased with burial depth. Emergence was reduced to 45% at 1–2 cm burial depths, and 0% at 8 cm. Emergence depths in concert with adequate moisture, germination across a range of temperatures, and rapid germination suggests *A*. *hispidus*’ seed bank may be short-lived in moist environments, but further investigation is warranted. Given the broad abiotic tolerances of *A*. *hispidus* and a widespread native range, *A*. *hispidus* has the potential to germinate in novel territories beyond its currently observed invaded range.

## Introduction

Sustainable invasive species management requires a thorough evaluation of foundational biology. Understanding germination, fecundity, morphology, growth physiology, dormancy, and competitiveness is important for quantifying economic and ecological impacts [1]. For example, plants with advantageous germination traits that promote establishment are more likely to become invasive [2]. These traits include but are not limited to, rapid germination, prolific seed production, and rapid growth [3]. Further, a species’ ability to proliferate may offer significant consequences to co-occurring native species in the form of competition and the modification of available resources such as light, water, and space [4].

*Arthraxon hispidus*, a novel invasive species in the Mid-Atlantic US, is an annual grass that utilizes the C4-photosynthetic pathway. It is classified with a wetland indicator ranging from facultative upland to facultative wetland [5]. Native to Asia, central-west Africa, and Australia, it was first observed in Pennsylvania, US in 1877 [6]. Nonnative populations have been observed spanning several countries in Central America and northern South America [7], which suggests an invasive potential that extends beyond the confines of the US.

Despite its long tenure in the United States, *A*. *hispidus* was only recently identified as an invasive species [7]. This lag phase often occurs from a lack of genetic variation, or dispersers, or because the population size has not reached a point that allows it to spread effectively [8]. However, populations of *A*. *hispidus* are found widespread across the eastern United States. This species is capable of high propagule pressure through the production of large quantities of seed coupled with clonal reproduction through the lower nodes of the culm [9]. When this species senesces, the plant material persists over multiple seasons resulting in a thick layer of thatch. Thatch is known to reduce forb performance and light availability which instigates a positive feedback loop for invasive grasses [10, 11]. The extent of the environmental impacts of *A*. *hispidus* is unknown in its invaded range and warrants further investigation.

Invasive grasses alter ecosystem dynamics in significant ways. In some cases, invasive grasses increase fire occurrence and frequency [12]. In others, they were shown to alter important microbial communities and plant-soil feedback systems which degrade native habitat and impede the reestablishment of native species [13, 14]. It is important to mitigate landscape spread early to prevent long-term consequences of invasive species establishment in novel territories [15].

Given its wide distribution both in its native and nonnative range [7, 16], it’s likely that *A*. *hispidus* is tolerant of a wide range of environmental filters. It is commonly observed in a variety of habitats such as roadsides, stream banks, ditches, fields, and disturbed habitats [9], but its biology is poorly understood. Seed germination is a critical life stage of plants and is influenced by several environmental factors including temperature, drought and salt stress, pH, and burial depth [17]. We designed a series of experiments using multiple populations of *A*. *hispidus* to better understand these environmental effects on seed germination, a critical life stage. With this information, we will have a better understanding of its potential for landscape spread and the ability to advise sustainable management approaches to control current *A*. *hispidus* populations.

## Materials and methods

### Seed sources

Seed from six populations of *A*. *hispidus* were collected from September to November 2020 to represent spatial and population variation from Virginia, Maryland, and Missouri (Table 1). Three of the original six populations used for the temperature experiment were analyzed further through germination at different pHs, salinities, and osmotic potentials to assess environmental tolerances and possible spatial variation. However, it is important to note that these seeds were collected from the environment. Environmental conditions may impact the quality of seed, germinability, and possible spatial variation caused by variation in genetics or environmental conditions. Limitations in space and materials prevented the use of all six populations in every experiment. Three populations with the highest spatial variation were selected for further analysis. Seeds were stored in manilla envelopes in the lab at ambient room temperature (~23°C) and humidity for several months to allow for any potential after-ripening. After initial storage, the seeds were dried at 40% relative humidity in a desiccator, sealed in mason jars, and stored at 4°C prior to germination experiments. No permits were required to obtain seeds in public rights-of-way.

**Table 1:** County, state, and GPS coordinates of the sampled *A*. *hispidus* populations. The X indicates the populations tested for each of the listed environmental stressor.

County & State	GPS Coordinates	Temperature	NaCl	Osmotic Potential	Emergence	pH
Frederick, MD	39°22’21.36"N 77°29’5.64"W	X	X	X	X	X
Lincoln, MO	39°02′01.0″N 90°55′51.0″W	X	X	X		X
Clifton, VA	38°45’13.40"N 77°23’31.50"W	X				
Pittsylvania, VA	37° 5’26.52"N 79°24’9.00"W	X				
Salem, VA	37°16’21.72"N 80° 6’57.60"W	X				
Williamsburg, VA	37°19’5.88"N 76°41’41.28"W	X	X	X		X

### Germination procedure

Most of the germination trials of *A*. *hispidus* were conducted by placing 20 seeds evenly spaced in polystyrene boxes (10.95 × 10.95 × 3.5 cm, Hoffman Manufacturing) on two thicknesses of germination blotter paper (10 cm × 10 cm × 1 mm, Anchor Paper Co., PBB 44, St. Paul, MN) and treated with approximately 14 mL of solution. To test the effects of pH on germination, seeds were incubated on Whatman #1 filter paper (47-mm diameter) and saturated with approximately 6 mL of solution instead of the germination blotter paper. Filter paper avoided potential changes in pH caused by germination blotter paper. The filter or blotter papers for germination experiments were fully saturated with either deionized water or a treatment solution and no excess solution pooled on the medium surface. One polystyrene box containing 20 seeds represented an experimental unit in each replication block. Each replication block was fully factorial containing every population at every level for each experiment. The pH and osmotic stress experiments were conducted at 23°C in an incubator. The NaCl and emergence experiments were conducted at temperatures of 23°C on the lab bench. Temperature treatments were conducted on a thermal gradient table (Thermogradient Systems LLC, Blacksburg. VA) [18]. The table was adjusted to provide a temperature gradient between two extreme temperatures and covered. Germination was measured by the protrusion of a 2-mm radicle. Germination was counted every 24 hours until seeds ceased to germinate for seven consecutive days unless stated otherwise. Seeds that germinated were removed each day. The germination percent, mean time to germination (MTG) and germination rate were calculated. The MTG is equal to Σ (N_i_ × T_i_) / (N) where is N_i_ the number of newly germinated seeds at time T_i_ recorded as days after imbibition. Germination rate was calculated by taking the reciprocal of MTG [19].


$$\text{MTG}=\Sigma \;\left(\text{Ni}\;\times \text{Ti}\right)/\Sigma \left(\text{Ni}\right)$$

GerminationRate=1/MTG

The mean minimum or base temperatures (T_b_) for seed germination were determined by extrapolating plots of mean GR verses temperature (T) to the intercept on the abscissa where the germination rate is zero.

### Temperature

Germination percentage and rate were recorded on blotter paper in sealed plastic boxes. Germination performance was assessed at 18 temperatures on a one-dimensional thermal gradient table as described above [18, 20, 21]. Cool temperatures (8.0, 11.3, 13.5, 14.5, 16.3, 18.5, 19.5, 21.3, 23.5) and warm temperatures (23.4, 24.0, 25.6, 27.2, 28.5, 30.0, 32.0, 34.0, 37.3) were run separately due to space limitations. The ranges overlapped at a temperature of 23°C to act as a control. Six populations were tested in each lane and replicated three times at each temperature (Table 1). Germination was recorded daily for the first 30 days and every two days from 30 to 60 days. The temperature was measured by placing three IButton thermochrons (IButton Link) wrapped in parafilm (Bemis Company, Neenah, WI) per isothermal lane inside the plastic boxes. The temperature was recorded every hour and averaged at the end of the trial. To mediate the differences in seed quality and better understand the species’ relationship with temperature as a whole, the germination percentage data was averaged.

### NaCl & osmotic potential

The effect of salinity on germination was in 0, 85, 171, 256, 342, 427, 513, and 599 mM NaCl. This range is congruent with concentrations in parts per thousand (0, 5, 10, 15, 20, 25, 30, 35 ppt) and represents a gradient ranging from freshwater to brackish and saltwater. The NaCl solutions were prepared by dissolving NaCl into deionized water.

In a separate experiment, water potentials were generated by dissolving inert nonpenetrating osmoticum (polyethylene glycol (PEG) 8000, Research Products International) in deionized water [22]. The water potential of osmotic solutions was verified by osmometry (Wescor 2500, Logan, UT). A gradient of osmotic potentials (0, -0.25, -0.47, -0.83, -1.2, and -1.46 MPa) were tested in boxes placed in self-sealing plastic bags to reduce evaporative losses.

### Depth to emergence

We tested the effect of burial depth on emergence using the Frederick, MD population. This population was chosen because it expressed the highest vigor across experiments. Fifty seeds were planted in plastic pots (15 × 15 × 13 cm) filled with clay loam topsoil collected from Salem, VA. Emergence was measured at 5 soil depths of 1, 2, 4, 6, and 8 cm. The pots were watered every day with approximately 250 mL to maintain adequate moisture. Emergence was determined by the protrusion of the cotyledon above the soil surface. The soil medium was autoclaved before planting to reduce microbial interactions. The experiment was conducted on the benchtop in the lab at an ambient temperature of ~23°C.

### pH

The effect of pH on germination was tested by preparing buffer solutions of 5, 6, 7, 8, 9, and 10 according to Chachalis and Reddy (2000). A 2-mM solution of MES [2-(N-morpholino)ethanesulfonic acid] was modified to pH 5 or 6 using 1 N hydrogen chloride (HCl) or sodium hydroxide (NaOH). A 2-mM solution of HEPES [N-(2-hydroxymethyl) piperazine-N-(2-ethanesulfonic acid)] was modified to pH 7 or 8 with 1 N NaOH. A pH 9 or 10 was prepared with a 2-mM tricine [N-Tris(hydroxymethylglycine] and raised with 1 N NaOH [23]. pH was verified before conducting the experiment using a pH meter (Mettler-Toledo LLC, Columbus OH).

### Statistics

Each experiment was organized into a randomized complete block design with three blocks of fully-factorial groups of experiment units referred to as replications. Regression analysis was conducted using SigmaPlot (version 14; Systat Software Inc.) and JMP Pro (version 16; SAS Institute). Constant temperature germination percent and rate data were analyzed with 3-segmented and linear regression, respectively. Germination rate data for osmotic potential and salinity were fitted with a polynomial regression model to evaluate treatment effects on *A*. *hispidus* germination. A Shapiro-Wilks test confirmed normality of the model residuals.

A functional three-parameter logistic model was fitted to the germination data measured in the osmotic stress and salinity experiments. G (%) represents germination percentage, G_max_ represents maximum germination percent, X_50_ is the value that inhibits 50% of the maximum germination (i.e. inflection point).


$$\text{G}\left(\%\right)={\text{G}}_{\text{max}}/[1+{\left(\text{X}/{\text{X}}_{50}\right)}^{\text{Slope}}]$$

To analyze the effects of burial depth, we fitted an exponential decay curve to the emergence data. E (%) is the emergence percent. E_max_ represents maximum emergence percent, the slope is the decay rate, and X is the burial depth (cm).


$$\text{E}\left(\%\right)={\text{E}}_{\text{max}}\times {\text{e}}^{\left(-\text{Slope}\times \text{X}\right)}$$

## Results and discussion

### Temperature

**Fig 1:**
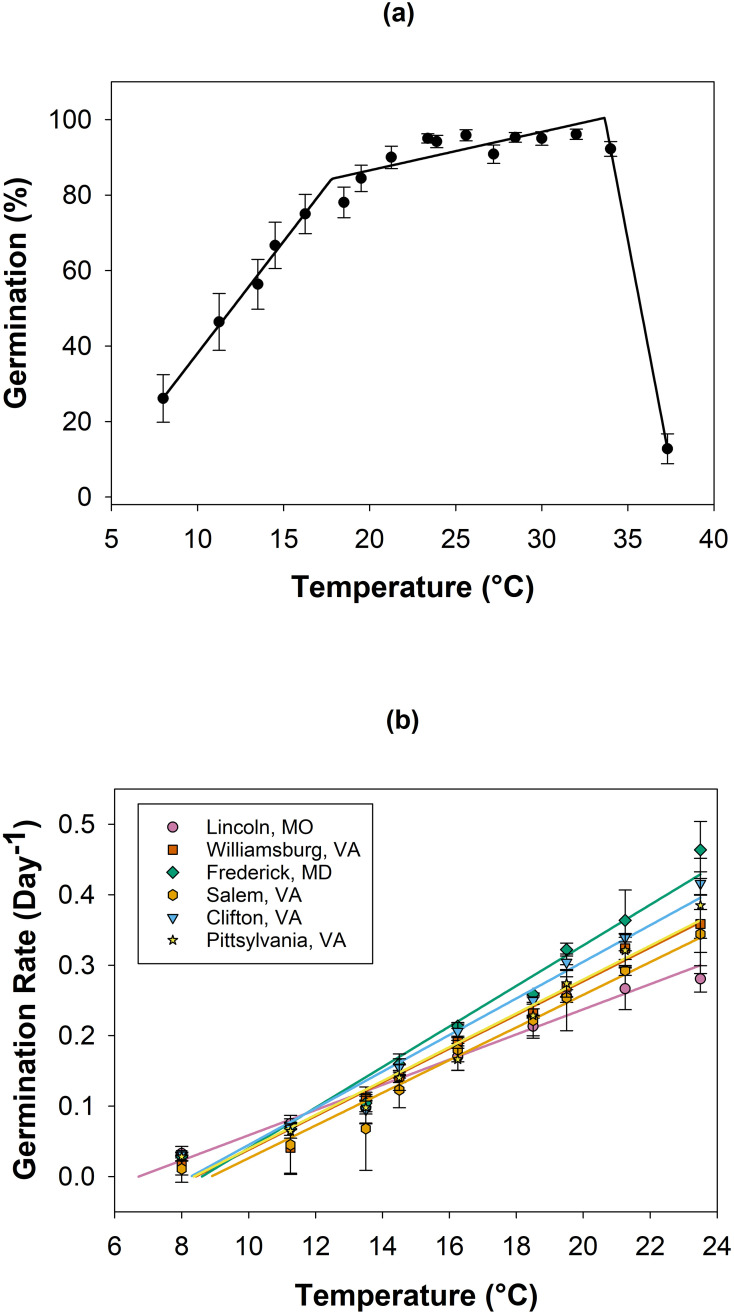
Constant temperature effects on germination percent data were analyzed using a segmented regression (a) and germination rate data were analyzed using linear regression (b) for 6 populations of *Arthraxon hispidus* (Table 1). The data points represent the mean ± standard error (SE).

Averaged across six populations, *A*. *hispidus* had >80% germination from 18 to 34°C. The highest temperature to decrease germination by 50% was 35.3°C (Fig 1a). The theoretical base temperature (T_b_) was calculated using the linear regression model (R^2^ = 0.94) according to Arnold (1985). T_b_ ranged from 6.7–8.9°C with the lowest temperature recorded from Lincoln, MO at 6.7°C. However, the other populations of *A*. *hispidus* exhibited less variation in based temperature and ranged from 8.2–8.9°C (Fig 1b).

The germination rate model (R^2^ = 0.94) determined a significant difference among populations (P < 0.0001, F-ratio = 13.39), temperature (P < 0.0001, F-ratio = 2567.41), and their interaction (P < 0.0001, F-ratio 9.60). As the temperature decreased, the germination rate also decreased. The Tukey’s honestly significant differences (HSD) post-hoc test determined the Frederick, MD population germinated at the highest rate (0.22 day^-1^) while the Salem, VA germinated at the lowest rate (0.17 day^-1^). Frederick, MD germinated at a higher rate than all populations except Clifton, VA. The only other significant difference measured was contributed to Frederick, MD and Clifton, VA significantly differing from Salem, VA and Lincoln, MO (Fig 1b).

The slopes of the regression lines in Fig 1b are the reciprocal or inverse of the thermal times to germination (1/θ_T_) allowing for a comparison of the speed at which different seed populations progressed toward germination at different temperatures. Variation in θ_T_ was observed among all populations. This variation undoubtedly affected the graphic estimates of T_b_. Since seed populations were harvested at different times and locations, physiological age of seeds likely varied greatly among locations and with harvest times. The slowing of germination rate is a classic sign of seed aging which may have occurred in some locations since no attempt to made to standardize seed development at the time of harvest. Thus, Fig 1b suggests that variation in seed quality may have affected germination performance in this study and is an alternative explanation to possible genetic differences in germination performance among the diverse populations. Replicates that experience >50% germination often underestimate the mean time to germination which predicts a faster germination rate. The rate measurements for these replicates represent the most vigorous fraction of seeds. Less vigorous seeds exposed to similar stress levels will germinate slower if not quiescent or nonviable [23, 24].

Comparing seed populations over large geographic areas is problematic since harvesting seeds at optimum maturity for the best seed quality is often impossible. A delay in harvesting may cause an abrupt transition from seed development to aging thus decreasing slopes (Fig 1b). Decreasing slopes result from slowing germination often resulting from aging [25, 26].

*Arthraxon hispidus* germinates across a range of constant temperatures (8–37°C). Temperature adaptations such as this may provide *A*. *hispidus* a competitive advantage during the growing season by germinating sooner and allocating resources earlier, which may impact biodiversity spanning several regions of the US. While this not only adversely affects native biodiversity but could also impact agriculture by reducing livestock forage in pasturelands [27].

When comparing constant temperature germinability to a common grass species in the US, southern crabgrass (*Digitaria ciliaris*), germination occurs across a nearly identical temperature range [28]. The distribution range of *A*. *hispidus* and *D*. *ciliaris* overlaps in many areas of the eastern and southeastern US. However, *D*. *ciliaris* is commonly observed in the southern and midwestern US in areas absent of *A*. *hispidus* invasion [9, 16]. The similarity in these two species may posit a larger invasive range for *A*. *hispidus*.

*Arthraxon hispidus* populations are distributed as far north as Massachusetts and as far south as Louisiana and encompass three prominent temperature regimes found in the US: mesic, thermic, and hyperthermic [16, 29]. The ability to germinate at cooler temperatures may present a competitive advantage in early-season acquisition of resources such as space, light, and water [4]. This trait could negatively impact desirable species in both native and managed ecosystems; however, research is required to quantify the extent of those impacts.

The significant difference in populations for the germination rate of *A*. *hispidus* could provide evidence for spatial genetic variability. The Lincoln, MO population’s T_b_ is 6.9°C which is cooler than the other populations. Since the Lincoln, MO population is geographically isolated from the other populations tested, variation in T_b_ suggests potential genetic variation between the populations [19, 21]. Aside from the Lincoln, MO population, T_b_ was similar and typical of the species as shifts in T_b_ are not associated with aging [25]. To further support spatial genetic variation among populations, lower germination percentages and rates were measured for the Salem, VA population as temperature decreased to 15°C.

### NaCl

**Fig 2:**
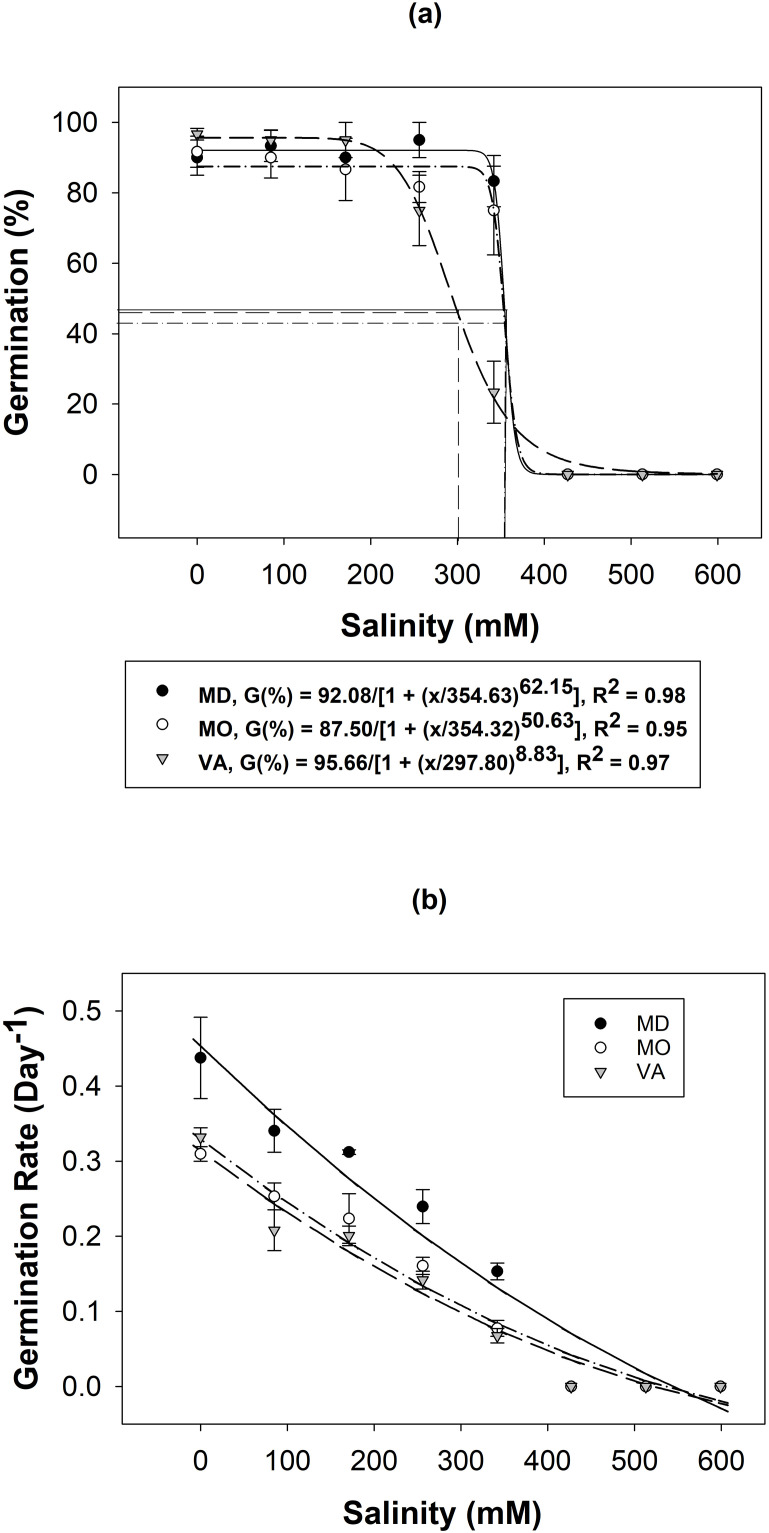
A three-parameter logistical regression (a) and a polynomial regression (b) modeling the effects of sodium chloride on *A*. *hispidus* germination using seeds sampled from Frederick, MD, Lincoln, MO, and Williamsburg, VA (Table 1). Error bars and lines represent ± SE and inflection points, respectively.

Salinity negatively impacted both the germination percent and germination rate of all three populations. Germination was >80% up to 196 mM NaCl for all populations. The Williamsburg, VA (VA) population slowly declined once it reached 256 mM NaCl while the others sharply declined at 344 mM NaCl. A 50% reduction in germination occurred (NaCl_50_) at 298 mM NaCl for VA and 354 mM NaCl for Fredrick, MD (MD) and Lincoln, MO (MO). No germination occurred at or beyond 427 mM NaCl (Fig 2a).

Salinity negatively impacted the germination rate of *A*. *hispidus*. The germination rate model (R^2^ = 0.94) indicated a significant difference between populations (P < 0.0001, F-ratio = 28.74), salinity levels (P < 0.0001, F-ratio = 1076.00) and their interaction (P < 0.0001, F-ratio = 15.42). The germination rate decreased from a range of 0.31–0.45 day^-1^ to 0.08–0.13 day^-1^ as NaCl concentrations increased from 0 to 342 mM NaCl. The Tukey’s HSD test determined the MD rate was significantly higher than both the VA and MO populations, which were significantly different from each other. The MD population germinated at higher rates across every salinity level (Fig 2b).

Salinity did not inhibit germination until it reached levels far beyond what is commonly observed in US soils (0–40 mM NaCl) [30]. Furthermore, germination was far more successful at higher salinity concentrations when compared to several other invasive species. Green galenia (*Galenia pubescens*), a noxious weed in Australia, had 50% germination at 115 mm NaCl and complete inhibition at 180 mm NaCl [31]. *Phalaris* spp. such as *P*. *minor*, *P*. *paradoxa*, and *P*. *brachystachys* had NaCl_50_ at lower levels of 35, 11, and 37 mM NaCl, respectively [32]. Lastly, *Phragmites australis*, a notorious saltmarsh invader, observed an 80% reduction in germination at concentrations starting at as little as 50 mM NaCl [33], which is substantially lower than germination percentages observed by *A*. *hispidus* at that concentration.

This higher tolerance to salinity in *A*. *hispidus* could facilitate invasion in systems commonly exposed to salt stress such as roadsides. Road salt pollution coupled with increased moisture from drainage ditches could exacerbate *A*. *hispidus* invasion along roadsides. In fact, *A*. *hispidus* is commonly observed along roadsides subjected to deicing salt (NaCl) during the winter months [9] and shares a similar salinity tolerance to the pervasive common ragweed (*Ambrosia artemisiifolia*), a notorious roadside invader [34].

This degree of salinity tolerance may also allow successful *A*. *hispidus* germination in sensitive saltmarsh ecosystems. The tested gradient represents salinity concentrations found in the environment ranging from brackish (8.5–513 mM NaCl) to marine systems (≥513 mM NaCl). Germination occurred at common saltmarsh concentrations (308–513 mM NaCl) [35]; which supports the potential of *A*. *hispidus* colonization in saltmarshes. However, the germination rate is significantly reduced as salinity concentration increases which may nullify the competitive advantage of rapid germination. More research is required to fully understand the invasion potential in saltmarshes as other life stages may not tolerate salinity. It is important to note that the MD and VA populations were collected along roadside ditches which are exposed to salt treatments during the winter months. However, the roads were not treated with salt in the fall when seed was collected.

### Osmotic potential

**Fig 3:**
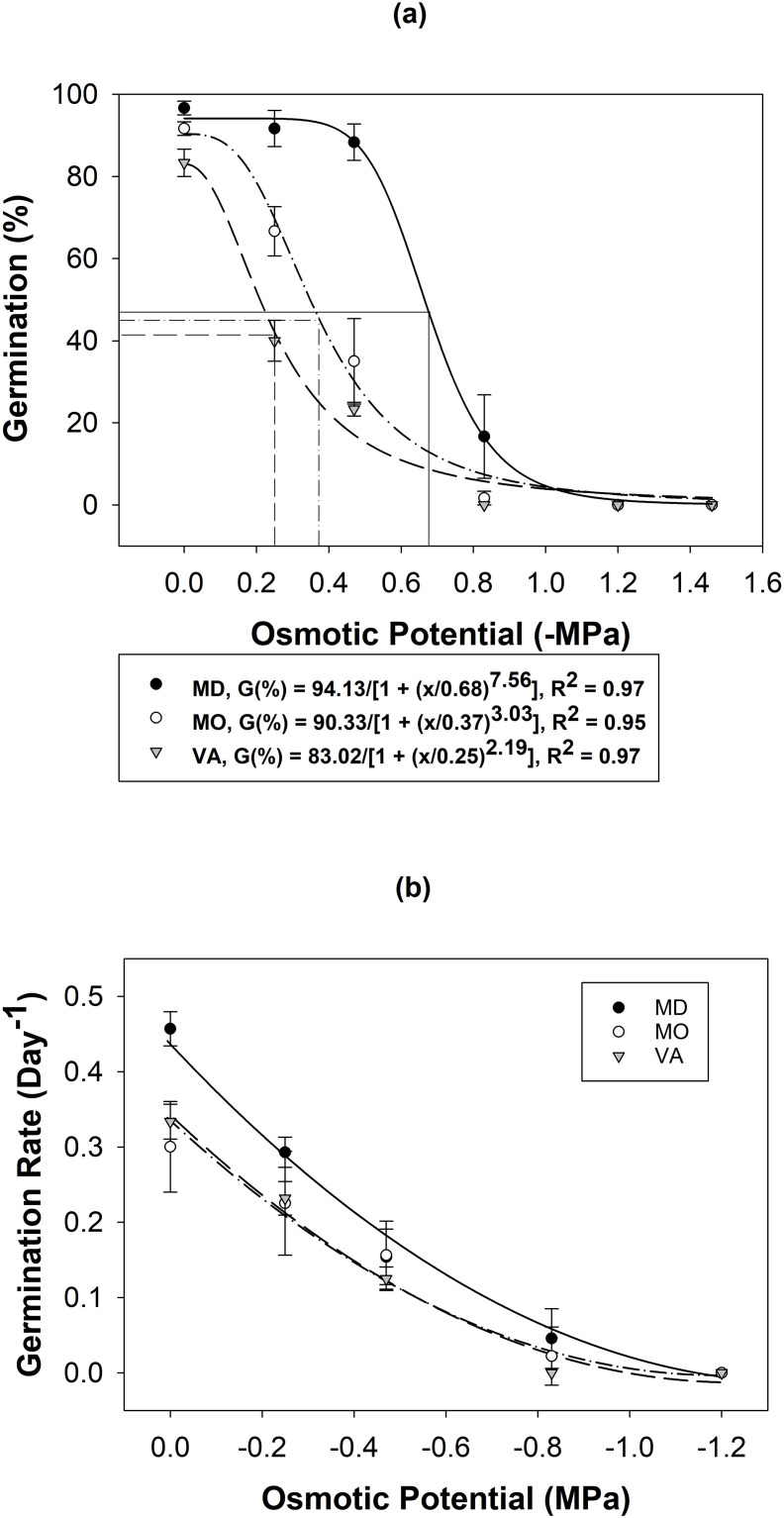
A three-parameter logistical regression (a) and a polynomial regression (b) modeled the effects of osmotic stress on *A*. *hispidus* germination using seeds sampled from Frederick, MD, Lincoln, MO, and Williamsburg, VA (Table 1). Error bars and lines represent ± SE and inflection points, respectively.

*Arthraxon hispidus’* germination was negatively impacted as osmotic potential decreased. Germination was reduced from ≥ 88% to ≤ 17% as osmotic potential decreased from 0 MPa to -0.83 MPa. At -0.83 MPa, MD, MO, and VA populations germinated 17%, 2%, and 0%, respectively, while no germination occurred ≤ -1.2 MPa. The osmotic potential required to inhibit germination by 50% (Ψ_50_) was -0.64 MPa for MD, -0.35 MPa for MO, and -0.23 MPa for VA (Fig 3a).

The germination rate of *A*. *hispidus* was negatively affected by osmotic potential. The germination rate model (R^2^ = 0.95) determined a significant difference in population (P < 0.0003, F-ratio = 9.74), osmotic potential (P < 0.0001, F-ratio = 911.10), and their interaction term (P < 0.0004, F-Ratio = 9.36). The germination rate decreased from a range of 0.34–0.44 day^-1^ to 0–0.06 day^-1^ as osmotic potential decreased from 0 to -0.83 MPa. The Tukey’s HSD test determined the MD population germinated at a significantly higher rate overall while MO and VA did not significantly differ from MD or each other. Maryland germinated at higher rates across every treatment level (Fig 3b).

This suggests that *A*. *hispidus’* germination can occur at lower osmotic potentials, but germination is most effective at higher moisture availability. While drought is a limiting factor for *A*. *hispidus* establishment [36], many of the observed populations are found within commonly distributed moisture regimes that likely maintain adequate soil moisture for extended periods of time throughout the growing season [16, 29]. Water availability is required for imbibition, embryo development, and seedling growth, so environments lacking adequate moisture will restrict germination. A reduction in germination at lower osmotic potentials could provide a fitness strategy to increase longevity in the seed bank until environmental conditions are conducive for survival [37].

On average, *A*. *hispidus*’ Ψ_50_ was lower when compared to the previous three *Phalaris* spp. (*P*. *minor*, Ψ_50_ = -0.08; *P*. *paradoxa*. Ψ_50_ = -0.25; *P*. *brachystachys*, Ψ_50_ = -0.32) [32]. However, *D*. *ciliaris* shared nearly identical germination patterns with *A*. *hispidus* across osmotic potentials [28]. Given *A*. *hispidus*’ affinity for moist conditions [9], upland areas with higher drainage classes and less precipitation will likely prevent germination. This is supported by the lower germination percentages found across the three populations as osmotic potential decreased (Fig 3a). The MD and MO population germinated at lower osmotic potentials (-0.83 MPa (17% and 2%)) which shows a potential to invade drier environments. However, drought can adversely affect *A*. *hispidus* establishment [36], so drought-prone environments are likely inhospitable for *A*. *hispidus* establishment.

### Depth to emergence

**Fig 4:**
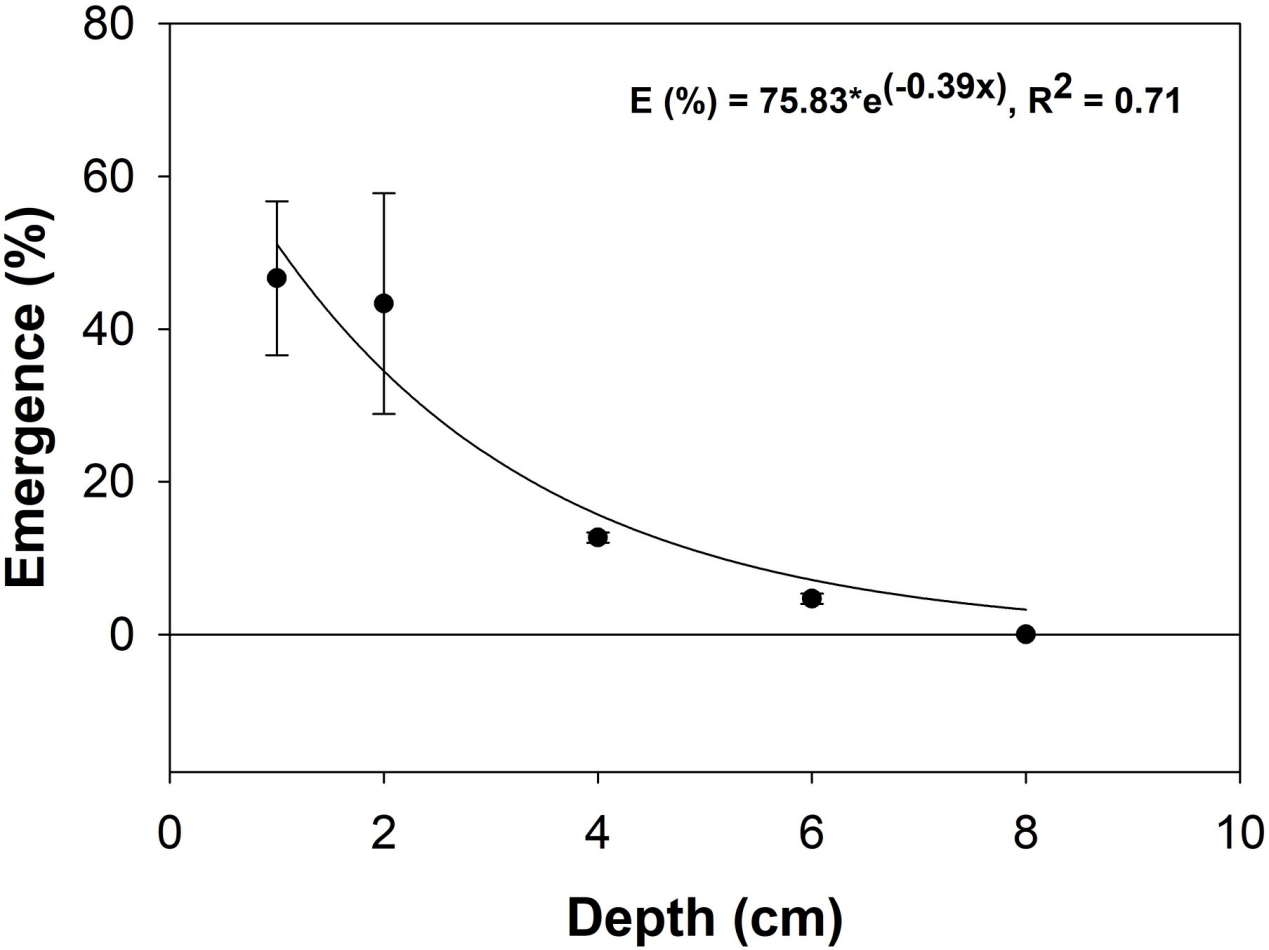
The effect of burial depth on *A*. *hispidus* emergence. An exponential decay model represents the curved line fitted to emergence data gathered using the Frederick, MD population (Table 1). Error bars represent ± SE.

Planting depth greatly reduced emergence percentages for the MD population. Emergence decreased from an average of 45% at 1–2 cm planting depth to 13% at 4 cm, and 5% at 6 cm (Fig 4). No seeds emerged at 8-cm depths. The model defined the E_max_ at 75.83% with a decay rate of -0.39 (R^2^ = 0.71).

While it is shown that *A*. *hispidus* can produce a viable seed bank within favorable conditions in Australia [38], the seeds tested in this study germinate at high rates in a wide range of environmental conditions which suggests the potential for limited seed-bank longevity... The level of effective germination across a gradient of environmental stressors suggests annual germination which could prevent seeds from persisting in the soil for multiple seasons. However, if seeds persist in the environment, the likelihood of translocation deeper into the soil profile increases, particularly with smaller seeds [39]. With decreasing emergence rates with depth, translocation to deeper soil horizons could negatively impact propagules. It could be disadvantageous for *A*. *hispidus’* seeds to persist in the environment for long periods of time. Granted, finer texture soils may produce higher emergence percentages [39]; therefore, the maximum emergence depth may depend on soil texture. It is possible seeds germinated at greater depths but the soil texture of the soil in this experiment may have limited emergence.

### pH

**Fig 5:**
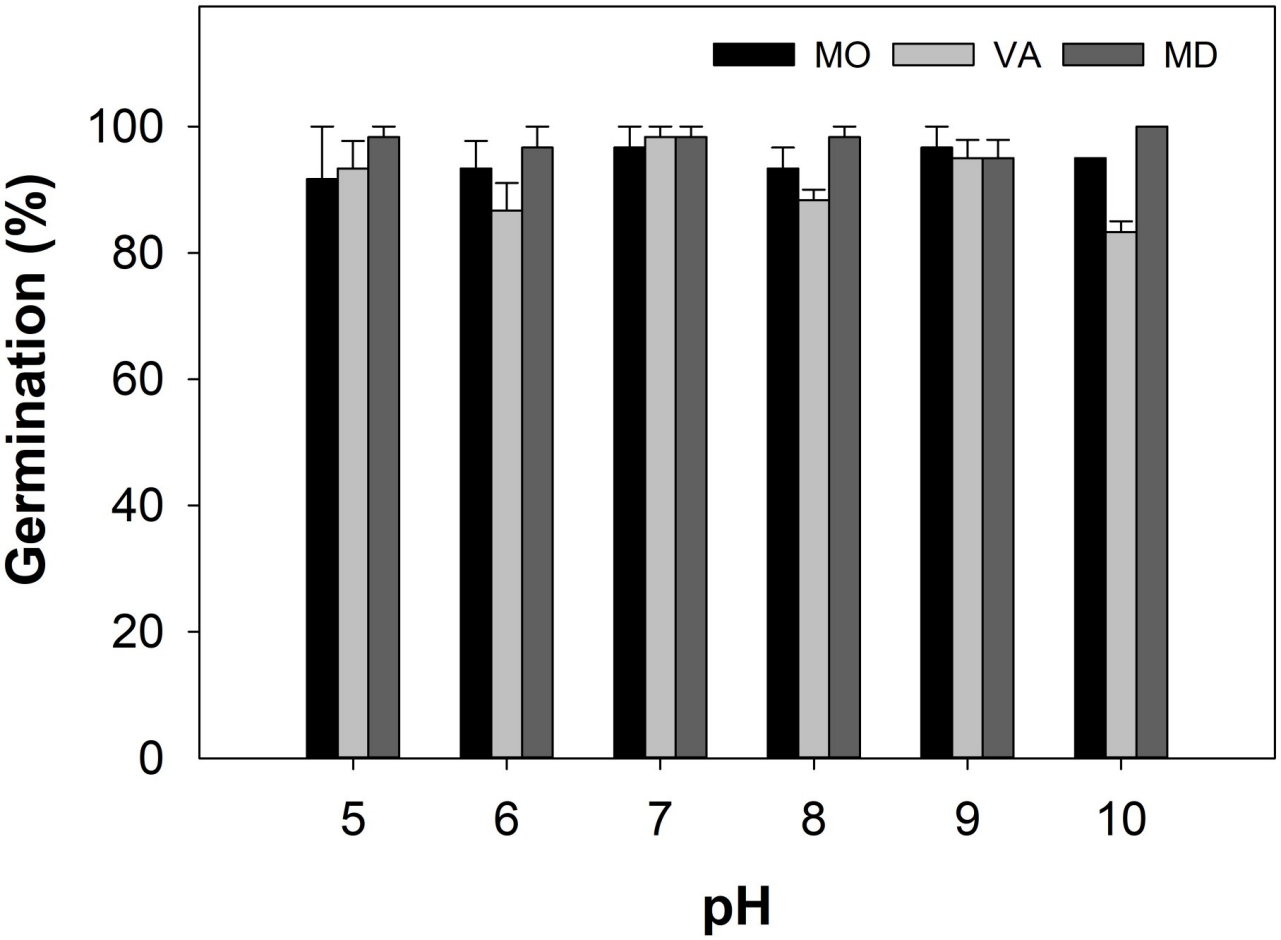
The effect of buffered pH solutions on *A*. *hispidus* germination from populations sampled in Frederick, MD, Lincoln, MO, and Williamsburg, VA (Table 1). Error bars represent ± SE.

Germination was ≥83% across the pH range of 5–10 for all populations tested (Fig 5). There were no significant differences across pH treatments. Germination percentages of this efficacy suggest a competitive advantage for *A*. *hispidus*’ establishment in both acidic and basic soil environments. This trait is commonly observed among weed species and offers increases invasiveness [40]. Soil should not prevent *A*. *hispidus* germination across a range of soil pH in the US [41].

## Conclusion

*Arthraxon hispidus* exhibits rapid and adaptable germination in a wide variety of environmental conditions. Consistent germination in stressful environments enhances its invasive potential, potentially in salt-stressed environments such as roadsides and tidal ecosystems. Although drought negatively affects germination, the species’ ability to germinate under diverse moisture conditions suggests the potential to overcome an initial life history barrier to population establishment in novel territories, especially in wetter years. Contrary to initial expectations, there is limited evidence to suggest geographic constraints to *A*. *hispidus* based on germination. To mitigate its impact, comprehensive research into the species’ foundational biology is essential for developing efficient control strategies and understanding its ecological ramifications at later life stages.

## Supporting information

S1 Data (XLSX)
